# Stimulation of de novo glutathione synthesis by nitrofurantoin for enhanced resilience of hepatocytes

**DOI:** 10.1007/s10565-021-09610-3

**Published:** 2021-05-22

**Authors:** Lukas S. Wijaya, Carina Rau, Theresa S. Braun, Serif Marangoz, Vincent Spegg, Matthijs Vlasveld, Wiebke Albrecht, Tim Brecklinghaus, Hennicke Kamp, Joost B. Beltman, Jan G. Hengstler, Bob van de Water, Marcel Leist, Stefan Schildknecht

**Affiliations:** 1 Division of Drug Discovery and Safety, Leiden Academic Centre for Drug Research, Leiden University, 2300 RA Leiden, The Netherlands; 2 In vitro Toxicology and Biomedicine, Department of Biology, University of Konstanz, P.O. Box M657, Universitätsstr. 10, 78457 Konstanz, Germany; 3 Konstanz Research School Chemical Biology (KoRS-CB), Department of Chemistry, University of Konstanz, 78457 Konstanz, Germany; 4 Leibniz Research Centre for Working Environment and Human Factors, Technical University Dortmund, Dortmund, Germany; 5 BASF SE, Experimental Toxicology and Ecology, Ludwigshafen am Rhein, Germany; 6 Albstadt-Sigmaringen University, Faculty of Life Sciences, 72488 Sigmaringen, Germany

**Keywords:** Nitrofurantoin, Hepatocytes, Nrf2, Glutathione, Cytochrome P450 reductase

## Abstract

Toxicity is not only a function of damage mechanisms, but is also determined by cellular resilience factors. Glutathione has been reported as essential element to counteract negative influences. The present work hence pursued the question how intracellular glutathione can be elevated transiently to render cells more resistant toward harmful conditions. The antibiotic nitrofurantoin (NFT) was identified to stimulate de novo synthesis of glutathione in the human hepatoma cell line, HepG2, and in primary human hepatocytes. In intact cells, activation of NFT yielded a radical anion, which subsequently initiated nuclear-factor-erythroid 2-related-factor-2 (Nrf2)-dependent induction of glutamate cysteine ligase (GCL). Application of siRNA-based intervention approaches confirmed the involvement of the Nrf2-GCL axis in the observed elevation of intracellular glutathione levels. Quantitative activation of Nrf2 by NFT, and the subsequent rise in glutathione, were similar as observed with the potent experimental Nrf2 activator diethyl maleate. The elevation of glutathione levels, observed even 48 h after withdrawal of NFT, rendered cells resistant to different stressors such as the mitochondrial inhibitor rotenone, the redox cycler paraquat, the proteasome inhibitors MG-132 or bortezomib, or high concentrations of NFT. Repurpose of the antibiotic NFT as activator of Nrf2 could thus be a promising strategy for a transient and targeted activation of the endogenous antioxidant machinery.

**Supplementary Information:**

The online version contains supplementary material available at 10.1007/s10565-021-09610-3.

**Figure:**
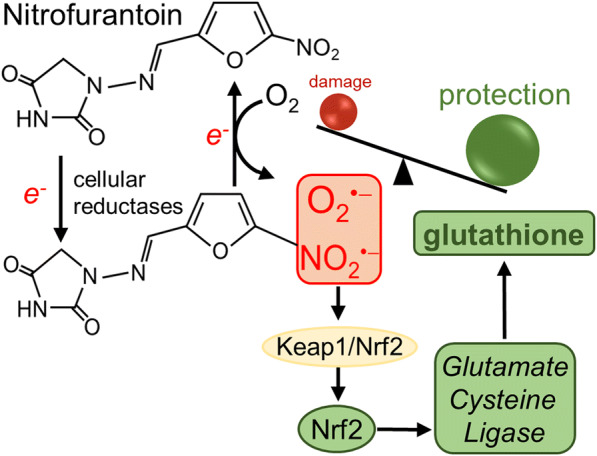
Graphical abstract

## Introduction

In its role as the principal detoxification organ, the liver is highly exposed to environmental toxicants, pharmacological compounds, and dietary ingredients. A large variety of these substances, either directly or in the course of their activation, contributes to the generation of oxidative stress. Systematic integration of quantitative and qualitative information into the framework of an adverse outcome pathway revealed the causal involvement of oxidative stress in liver pathologies such as drug-induced liver injury, alcoholic liver disease, cholestatic liver injury, and nonalcoholic fatty liver disease (Kuijper et al. 2017; Khadka et al. 2020; Horvat et al. 2017; Vinken et al. 2013). Many approaches have been attempted in order to alleviate or prevent liver disease via the reduction of oxidative stress (Li et al. 2015). Unfortunately, none of these strategies have been successful. Treatment of patients with small-molecule antioxidants or antioxidant enzymes did not yield the desired benefits (Lirussi et al. 2007; Bjelakovic et al. 2011, 2017). Indeed, treatment with relatively high doses of antioxidants can even be linked to potential adverse effects due to interference with reactive oxygen and nitrogen species involved in normal cellular signaling (Frein et al. 2005; Schildknecht et al. 2009a).

To avoid these limitations, targeted activation of the endogenous cellular antioxidant machinery has emerged as an alternative therapeutic strategy for the reduction of oxidative stress (Davis and Pennypacker 2017; Kang and Kang 2013). In this context, several sensors and pathways regulating the transcription factors, such as activator protein 1 (AP-1), cAMP response element-binding protein (CREB), hypoxia inducible factor-1 (HIF-1), nuclear factor κB (NF-κB), activating transcription factor 4 (ATF-4), and nuclear factor-erythroid 2-related factor-2 (Nrf2), have been described (Marinho et al. 2014; Cuadrado et al. 2018). These transcription factors are involved in the synthesis of several antioxidant enzymes (e.g., glutathione reductase, glutathione peroxidases, glutaredoxin, and glutathione-*S*-transferase), many of which are closely related to the cellular antioxidant, glutathione (Espinosa-Diez et al. 2015). Glutathione represents the most abundant intracellular antioxidant capable of (i) directly interacting with free radical species, (ii) revitalizing other small molecule antioxidants (e.g., vitamins C or E), (iii) maintaining the redox state of protein thiols, and (iv) serving as a cofactor for enzymes of the cellular antioxidant system (Espinosa-Diez et al. 2015). Glutathione-mediated redox regulation is determined not only by the ratio of its reduced (GSH) and oxidized (GSSG) forms but also by their absolute quantities in a cell. De novo glutathione synthesis is catalyzed by the heterodimeric glutamate cysteine ligase (GCL), which is composed of catalytic (GCLC) and modifier (GCLM) subunits (Lu 2013). GCL-catalyzed conjugation of the γ-carbonyl group of glutamate with the amino moiety of cysteine is the rate-limiting step in glutathione synthesis. The catalytic subunit of GCL is subject to competitive feedback inhibition by glutathione, thus preventing an excess of glutathione production (Richman and Meister 1975). In a second enzymatic step, glutathione synthetase catalyzes the condensation of γ-glutamylcysteine and glycine to form the tripeptide, glutathione.

The synthesis of GCL, along with other targets involved in the antioxidant response, is under the control of the transcription factor, Nrf2 (Baird and Dinkova-Kostova 2011). Under normal conditions, Nrf2 is anchored in the cytosol through its interaction with Kelch-like ECH-associated protein 1 (Keap1), which serves as a substrate adapter protein for an E3 ubiquitin ligase complex (Zhang et al. 2004). Because of ubiquitination and subsequent proteolysis, Nrf2 turns over rapidly with a half-life of 7–15 min, which increases to 30–100 min in the presence of oxidative or electrophilic stress (McMahon et al. 2004; Nguyen et al. 2003). Cysteine residues in Keap1 act as sensors for electrophiles and oxidants, and their oxidation leads to the release and subsequent translocation of Nrf2 into the nucleus where it can bind to the ARE4 sequence in the promoter region of genes involved in antioxidant defense (Moinova and Mulcahy 1999).

Observations indicating a parallel, opposing influence of Nrf2 on the transcriptional upregulation of pro-inflammatory genes suggest that targeted pharmacological activation of the Nrf2 pathway could help in the controlled reduction of oxidative stress (Kobayashi et al. 2016). Besides well-known oxidants or electrophiles, such as H_2_O_2_, 4-hydroxynonenal, and diethyl maleate (DEM), which also possess cytotoxicity, only few Nrf2 activators have been found to be useful in clinical trials (Sun et al. 2017) (clinicaltrials.org). Oleanolic acid-derived bardoxolone methyl, a dual activator of Nrf2 and inhibitor of the NF-κB pathway, entered clinical trials for the treatment of diabetic nephropathy and chronic kidney disease (de Zeeuw et al. 2013). It was withdrawn during phase 3 due to heart failure problems, but is still in clinical development for alternative indications. Dimethyl fumarate is currently the only Nrf2 activator with FDA approval for the treatment of multiple sclerosis but is also associated with desensitization and other side effects (Gold et al. 2012; Xu et al. 2015). Along with alternative approaches, such as protein-protein interaction inhibitors (Richardson et al. 2015), or modulators of Nrf2 phosphorylation (Chowdhry et al. 2013), the vast majority of potential Nrf2 modulators are electrophiles with an inherent propensity to interact with off-target cellular nucleophiles.

The present study hence focused on compounds, already in clinical use, with the potential to activate the Nrf2 stress response pathway. Nitrofurantoin (NFT) demonstrated Nrf2 activation and glutathione synthesis stimulation comparable to the potent experimental Nrf2 activator diethyl maleate. These effects were observed at NFT concentrations reflecting NFT levels reported in human plasma following standard oral treatments when applied in its role as antibiotic.

## Material and methods

### Cell culture

Human hepatocellular carcinoma (HepG2) (ATCC, HB-8065) were maintained in DMEM (4.5 g/l glucose, pyruvate) (Gibco), supplemented with 10% fetal bovine serum (PAA Laboratories) and penicillin/streptomycin (25 μg/ml each, Gibco) at 37 °C, 5% CO_2_. Cells were seeded at a density of 60,000 cells/cm^2^ and allowed to proliferate for 3 days. Following medium change, cells were treated as indicated. Cells were routinely (6 times per year) tested for mycoplasma contamination.

Primary human hepatocytes (PHH) were obtained from BioIVT (donors IAN, IPH, GID) and from Lonza (donors HUM4108, HUM4055B, HUM4229, HUM181501B). Cells were seeded on collagen-coated plates (250 μg/ml rat collagen, Roche) at a density of 150,000 cells/cm^2^ and cultivated for 3 h in William’s E medium (PAN Biotech) containing penicillin (100 U/ml, Gibco), streptomycin (0.1 mg/ml, Gibco), gentamycin (10 μM, PAN Biotech), dexamethasone (100 nM, Sigma), stable l-glutamine (2 mM, Sigma), insulin supplement (2 ng/ml, Sigma), and 10% Sera Plus (PAN Biotech). After 3 h for cell attachment, medium was exchanged to Sera Plus-free medium and cells were allowed to adjust for 16 h before treatment.

### Generation of GFP-tagged cell lines

HepG2-GFP reporter cell lines expressing tagged Nrf2, HMOX1, or SRXN1 were constructed with bacterial artificial chromosomes that encode C-terminal GFP-tagged fusion proteins as described previously (Wink et al. 2014, 2017, 2018).

### Live cell image processing

Translocation of Nrf2-GFP to the nucleus and accumulation of HMOX1-GFP and SRXN1-GFP expression was monitored by a Nikon TiE2000 confocal laser microscope equipped with an automated focus system. After Hoechst H-33342 nuclei staining, NFT or diethyl maleate (DEM) were added for up to 60 h, followed by automated live cell confocal imaging. High content image analysis pipelines were applied to quantify cellular responses as described previously in detail (Wink et al. 2014, 2017, 2018).

### Cell viability

#### Resazurin reduction assay

Resazurin (Sigma) was added to the cell culture medium (5 μg/ml) for a period of at least 30 min. Resorufin fluorescence was determined at 530 nm_ex_/590 nm_em_ with a Tecan Infinite M200 reader. Cell viability was expressed as percentage of fluorescence intensity relative to untreated controls. Background fluorescence was determined in each individual experiment with cells exposed to Triton (1%) and subtracted from all other values.

#### Lactate dehydrogenase release assay

Lactate dehydrogenase (LDH) activity was determined in the cell homogenate and in the corresponding supernatant, respectively. Cells were lysed in PBS/Triton X-100 (0.5%) for 60 min. Cell homogenate and supernatant (10 μl) were transferred into a 96-well plate. The reaction was initiated by the addition of 190 μl reaction buffer, adjusted to pH 7.4 by titration with K_2_HPO_4_ (40 mM) and KH_2_PO_4_ (10 mM) stock solutions, supplemented with NADH (100 μM) and sodium pyruvate (600 μM). NADH consumption was detected at 340 nm, recorded in 1 min intervals over a period of 20 min. LDH release was expressed as ΔOD_340_ (supernatant) / ΔOD_340_ (supernatant + cell homogenate).

### Glutathione detection

Cells in 96-well plates were lysed by the addition of 100 μl of 1% sulfosalicylic acid. Lysed cells (20 μl) were transferred to a new 96-well plate and supplemented with 80 μl water. A standard curve of GSH was prepared in 1% sulfosalicylic acid in the range of 0.1 to 10 μM. The recycling reaction was initiated by the addition of reaction buffer (100 mM sodium phosphate, pH 7.4), supplemented with 5,5′-dithiobis (2-nitrobenzoic acid) (DTNB) (100 μM) (Sigma), NADPH (100 μM) (Roth), glutathione reductase (1 U/ml) (Sigma), and EDTA (1 mM) (Sigma). Total protein amounts were detected in parallel by the BCA assay (Pierce™ BCA Protein assay Kit) for normalization of glutathione levels. If not otherwise indicated, detection of glutathione includes reduced (GSH) and oxidized (GSSG) glutathione. For separate detection of the two forms, a portion of the sample was treated with 5% 2-vinylpyridine (Sigma) for 60 min to trap GSH. Both untreated samples (containing GSH and GSSG) and 2-VP treated samples (containing GSSG) were assessed by the DTNB assay in parallel.

### Nrf2 staining and translocation

For visualization and quantification of endogenous Nrf2 translocation into the nucleus, glass 96-well plates were coated with fibronectin (1 μg/ml) (Sigma) and poly-l-ornithine (40 μg/ml) over night. Plates were washed two times with water before use. HepG2 were seeded at a density of 10.000 cells/cm^2^. Following treatment, the medium was removed, cells were washed 2 times with warm PBS, and fixed with 4% formaldehyde for 20 min. Following three washing steps with PBS, cells were permeabilized with 0.1% Triton for 20 min and blocked with 2% BSA in PBS-Tween (0.5%) at room temperature for at least 1 h. The cells were then incubated with monoclonal anti-Nrf2 antibody (1:100, Santa Cruz sc-518033) at 4 °C, over-night. After five washing steps with PBS-Tween, the secondary antibody (Alexa Fluor 555 anti-mouse, Life Technologies, 1:200) was added in 2% BSA/PBS-Tween together with Hoechst 33342 (1 μg/ml) for 2 h. For visualization, an Olympus IX81 microscope, equipped with a F-view CCD camera was used. For quantitative assessment of Nrf2 translocation from the cytosol to the nucleus, an automated Cellomics ArrayScan (Thermo Fisher) microscope system was employed. Nuclei, stained with H-33342, were imaged first for automated focusing and identification of valid objects. Nrf2 was subsequently imaged in the corresponding areas. For assessment of the nuclear cytoplasmic ratio of Nrf2 signal intensity, the nucleus was defined by Hoechst H-33342 staining. The cytoplasmic area was defined as a ring region with a width of 1.9 μm and a distance of 3.3 μm from the nuclear outline.

### siRNA-mediated knockdown of Nrf2 and CYPOR

For transfection of HepG2 in (an individual well) of a 6-well plate (5 × 10^5^ cells/well), solution A, consisting of 4 μl Lipofectamine™ (Life Technologies) and 150 μl Opti-MEM® (Life Technologies) were mixed. Solution B consisted of 40 μl (Nrf2) respectively 80 μl (CYPOR) of a 10 μM siRNA stock solution, mixed with 150 μl Opti-MEM®. For an individual well of a 96-well plate, solution A consisted of 0.2 μl Lipofectamine™ and 5 μl Opti-MEM®; solution B consisted of 5 μl Opti-MEM® and 1.5 μl (Nrf2), respectively 3 μl (CYPOR) of a 10 μM siRNA stock solution. After an incubation period of 5 min at RT, both solutions were combined, incubated for 30 min. HepG2 (5 × 10^5^ cells/cm^2^) were washed two times with Opti-MEM® before the siRNA solution was added. After 24 h, an equal amount of DMEM containing 20% serum was added. Twenty-four hours (Nrf2), respectively 48 h (CYPOR) after seeding, treatment of the cells with NFT was initiated.

### Western blot

Cells were lysed in 1× Laemmli buffer and centrifuged at 10,000 *g* for 2 min through a Nucleo Spin filter (Machery Nagel). Lysates were boiled at 95 °C for 5 min, 20 μg of total protein were subjected to separation by a 10% SDS gel, transferred onto nitrocellulose membranes (Amersham Biosciences) and blocked with 5% milk powder in PBS Tween (0.1%) for 1 h. The following antibodies were used: anti-Nrf2 (monoclonal, 1:1000, Santa Cruz); anti-GCLC (rabbit, 1:1000, Bioworld Technologies); anti-GCLM (rabbit, 1:2000, Proteintech); anti-CYPOR (monoclonal, 1:500, Santa Cruz); HRP-conjugated anti mouse IgG (1:5000, Jackson Immuno Research); and HRP-conjugated anti-rabbit IgG (1:5000, Amersham). Protein bands were detected by a FUSION SL™ system (Peqlab, Erlangen, Germany) and quantified by ImaEva or ImageJ.

### EPR spectroscopy

Continuous wave EPR spectroscopy was performed at 20 °C with a X-band (9.6346 GHz) spectrometer (EMX-Nano, Bruker Biospin, with a cylindric cavity mode TM1110). Microsomes were prepared by sonication of 10^8^ cells, removal of debris by centrifugation at 10,000 *g* for 15 min, and subsequent centrifugation of the resulting supernatant at 100,000 *g* for 60 min. Microsomes (1 mg/ml) in sodium phosphate buffer, pH 7.4 were supplemented with NADPH (10 mM) and NFT (5 mM). For analysis of intact cells, HepG2 (10^7^/ml in DMEM medium w/o serum and antibiotics) were treated with NFT (5 mM). Samples were loaded into glass capillaries (Hirschmann® ringcaps®, 1 mm inner diameter) and sealed with Hemato-Seal™ capillary tube sealant (Fisherbrand™). A microwave power of 6.3 mW and a modulation amplitude of 5 G at a modulation frequency of 100 kHz were used to acquire spectra in the range of 3367 G to 3497 G at a sweep time of 156.22 s and a conversion time of 78.11 ms. Further, 117 or 12 scans were accumulated for the samples with microsomes or whole cells, respectively. All spectra were baseline-corrected using MatLab2019b and EasySpin 5.2.25 (Stoll and Schweiger 2006). Spectra were background-corrected by subtraction of the spectrum obtained in the presence of microsomes and NADPH, respectively intact HepG, but in absence of NFT.

### TempO-Seq transcriptome analysis

HepG2 cells or PHH were seeded in 96-well plates (20,000 cells/well) and exposed to different concentrations of NFT ranging from 5 to 125 μM. The plates were then washed with 1× PBS, and cells were lysed with 50 μl BioSpyder 1× lysis buffer for 15 min at RT. The plates were kept at – 80 °C until shipment. The frozen plates were shipped to BioSpyder Technologies. The Deseq2 package in R was employed for the calculation of log2 fold changes in transcript expression.

### Statistics

Values are expressed as mean ± SD. Experiments were performed at least three times with at least three technical replicates in each experiment. Differences were tested for significance by one-way or two-way ANOVA, followed by Bonferroni’s post hoc test, *p* < 0.05. If not otherwise indicated, differences between means were considered statistically significant at *p* < 0.05. Statistical differences were tested using GraphPad Prism 5.0 (GraphPad Software, La Jolla, USA).

## Results

### Elevation of intracellular glutathione levels by NFT

**Fig. 1:**
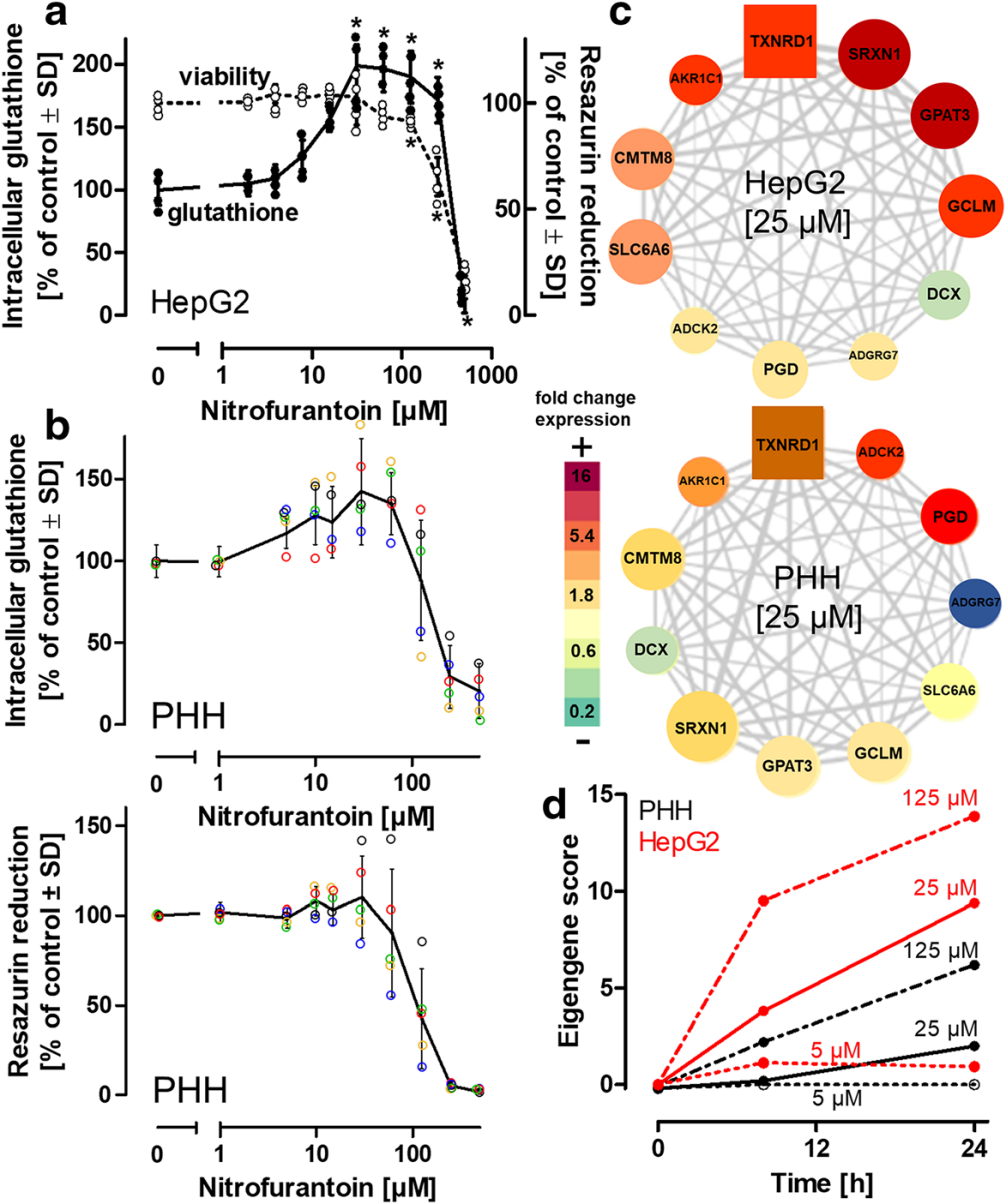
Elevation of glutathione by nitrofurantoin*.* **a** HepG2 (300,000 cells/cm^2^) were treated with indicated concentrations of nitrofurantoin (NFT). Intracellular levels of glutathione (reduced + oxidized) were detected after 48 h. Viability was assessed by measuring the reduction of resazurin. **b** Primary human hepatocytes (PHHs) from 5 donors were seeded at a density of 150,000 cells/cm^2^ and treated with NFT for 48 h. The colored circles represent values obtained with cells from 5 individual donors. The black solid lines represent the means of values from all five donors. Intracellular levels of glutathione (reduced + oxidized) were detected after 48 h. Viability was assessed by measuring the reduction of resazurin. **c** Gene co-expression analysis. Weighted gene co-expression network analysis was applied to the transcriptomic profiling of HepG2 exposed with NFT, utilizing the TXG-MAPr platform, allowing clustering of gene sets into functional modules. Significant changes in gene expression were identified for the module “oxidative stress” (module 144). The square node represents the gene with the highest correlation relative to the response of the entire module (hub gene). The size of the round nodes indicates the correlation of gene expression with the response of the entire module. The color code of the heatmap indicates up- or downregulation of the individual genes relative to their expression in untreated cells. **d** Eigengene scores indicating the sum of z-scores from the log2 fold changes of the individual genes of the module “oxidative stress” calculated in HepG2 or PHHs exposed for 8 h to NFT. Differences (in **a**; *n* = 5) were tested for significance by one-way ANOVA, followed by Bonferroni’s post hoc test, *p < 0.0*5* for comparison of treatments with the respective untreated controls. In (**b**), means ± SD are indicated by the solid line. Individual values obtained with cells from 4 different donors are indicated by colored dots. *PGD* 6-phosphogluconate dehydrogenase, *SLC6A6* solute carrier family 6 member 7, *CMTM8* CKLF-like MARVEL transmembrane domain containing 8, *ADCK2* AarF domain containing kinase 2, *ADGRG7* adhesion G protein-coupled receptor G7, *DCX* doublecortin, *GCLM* glutamate cysteine ligase modifier subunit, *AKR1C1* Aldo-keto reductase family 1 member, *TXNRD1* thioredoxin reductase 1, *SRXN1* sulfiredoxin 1, *GPAT3* glycerol-3-phosphate acetyltransferase 3

We had previously demonstrated the activation of cellular oxidative stress response pathways by NFT (Bischoff et al. 2019) (Suppl. Fig. 1). We subsequently identified NFT as a potent stimulator of intracellular glutathione synthesis in the human hepatoblastoma-derived HepG2 cell line (Fig. 1a). The observed increase peaked between 24 and 48 h of NFT treatment and culminated in the doubling of intracellular glutathione levels. Cell viability decreased significantly at NFT concentrations > 100 μM (Fig. 1a), accompanied by impaired mitochondrial respiration as one of the main adverse molecular events (Suppl. Fig. 2). At NFT concentrations < 100 μM, cell integrity was not affected even at incubation periods of up to 6 days (not shown), suggesting that 5–50 μM NFT could be used for targeted stimulation of endogenous glutathione synthesis.

In this study, glutathione detection reflects the sum of its reduced (GSH) and oxidized (GSSG) states, unless indicated otherwise. Individual detection of GSH or GSSG indicated a GSH:GSSG ratio of approximately 100:1 in untreated cells. Following NFT treatment at up to 50 μM for 48 h, GSSG levels increased by a factor of two (Suppl. Fig. 3a,b). However, due to the concurrent increase in GSH, the GSH:GSSG ratio remained almost identical (Suppl. Fig. 3c). Although GSH and GSSG were detected in the extracellular medium, no significant increase was observed upon incubation of cells with nontoxic NFT concentrations, thus excluding the possibility of elevated export of GSH and GSSG (Suppl. Fig. 3d).

Hepatocyte cell lines are considered poor representatives of essential hepatocyte features. Hence, primary human hepatocytes (PHHs) from 5 individual donors were employed as alternative model that is considered to closely resemble the situation in vivo. PHH were exposed to the same NFT treatment conditions as the HepG2 cells (Fig. 1b). The average glutathione levels in untreated PHHs were 20 nmol/mg protein and thus were comparable to the baseline levels in HepG2 (28 nmol/mg protein) (Suppl. Fig. 4). Similar to HepG2 cells, an increase in intracellular glutathione levels was observed in the PHHs for the same NFT concentrations of 5–50 μM. Although the primary cells displayed inter-individual differences (different ages and medical histories of the donors, Suppl. Fig. 5) in their response to NFT, cells from all donors showed an increase in glutathione levels following NFT treatment (Fig. 1b). To address any potential cell-type-dependence of the observed increase in glutathione levels, we examined differentiated post-mitotic neuronal LUHMES cells as an alternative model (Schildknecht et al. 2009b; Scholz et al. 2011). The cells were treated in an identical manner and exhibited a doubling of intracellular glutathione levels (Suppl. Fig. 6), whereas human iPSC-derived astrocytes exhibited an only marginal response (Suppl. Fig. 6). Furthermore, a correlation coefficient of > 0.5 was observed in a comparison of gene expression profiles of PHH and HepG2 when exposed to different NFT concentrations (Suppl. Fig. 7). These observations highlight the suitability of the HepG2 model to study these events and the necessity to define cellular sensors and signaling cascades involved in the NFT-induced increase of glutathione levels.

### Activation of Nrf2 by NFT

To gain insight into pathways that may be involved in the observed increase in glutathione levels, we conducted high throughput targeted RNA sequencing of samples from HepG2 cells. The results were uploaded to a pathway mapping platform (http://txg-mapr.eu) to identify the activated pathways. This platform was developed utilizing unbiased weighted gene co-expression network analysis (WGCNA) from PHH microarray data obtained from an open data repository, TG-GATES. The WGCNA approach, applied to this set of microarray data, clusters co-regulated genes based on their co-expression pattern and organizes them into “modules.” An eigengene score is thereafter applied to each module derived from the z-scored log 2-fold change values of the genes consisting the module (module membership). These eigengene scores indicate the modulation of the module in regards to cellular responses.

In both HepG2 cells (experimental data) and PHHs (data from TG-GATES), NFT revealed an activation of module 144, showing “oxidative stress response” as the module ontology (Fig. 1c). The eigengene score of module 144 indicates a concentration-dependent activation of gene expression by NFT (Fig. 1d). Among the module membership was GCL, composed of the catalytic GCLC and the modifier GCLM subunit, shown to be upregulated (Fig. 1c). This gene is known to catalyze the rate-limiting step in glutathione synthesis and thus emerged as a top candidate for the observed rise in glutathione.

**Fig. 2:**
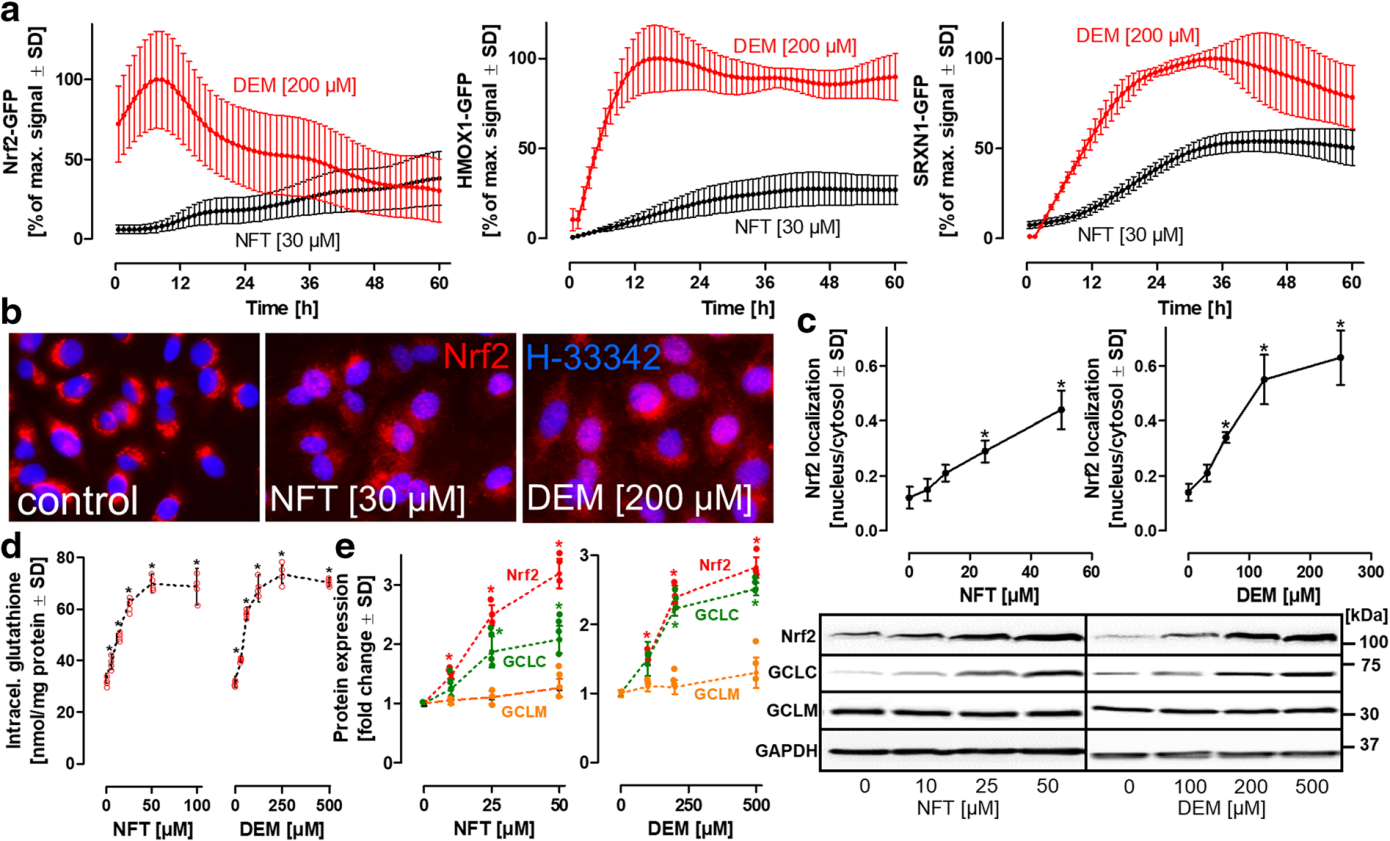
Dynamics of cellular oxidative stress response pathway activation. **a** HepG2 reporter cell lines, expressing green fluorescent protein (GFP)-coupled oxidative stress reporter elements, Nrf2 (nuclear factor erythroid 2-related factor 2), HMOX1 (heme oxygenase), or SRXN1 (sulfiredoxin), were exposed to NFT or to diethyl maleate (DEM) (positive control). Automated high-content confocal imaging and quantitative single cell image analysis were employed for time-resolved monitoring of Nrf2-GFP stabilization and nuclear translocation, as well as for the assessment of the subsequent induction of the downstream targets, SRXN1 and HMOX1. Values are expressed as percentages of the maximal GFP signal detected with DEM (positive control). **b** For visualization of endogenous Nrf2 stabilization and translocation, wild-type HepG2 cells were treated with NFT or DEM for 24 h, fixed, and stained with an anti-Nrf2 antibody (red). Nuclei were stained with Hoechst H-33342 (blue). **c** For quantitative assessment of endogenous Nrf2 translocation into the nucleus, a nuclear area and a corresponding cytoplasmic area were defined, and the sum of intensities for all Nrf2 pixels within these areas were used for the calculation of the nuclear/cytosolic ratio for each individual cell. Values are means of 6 independent experiments. In each experiment, a minimum of 1000 cells was analyzed. **d** Intracellular glutathione levels in HepG2 exposed to NFT or DEM for 24 h. Values are means of 4 independent experiments ± SD. Individual values are represented by red circles. **e** Protein levels in HepG2 exposed to NFT or DEM for 24 h. Western blotting with antibodies selective for Nrf2, glutamate cysteine ligase catalytic (GCLC), or modifier (GCLM) subunits. Protein levels were normalized to GAPDH levels. Protein levels of untreated controls were defined as unity, and protein levels were expressed in terms of fold change compared to controls. Values are means of 4 independent experiments ± SD. Individual values are represented by colored circles. Differences (**c**, **d**) were tested for significance by one-way ANOVA, followed by Bonferroni’s post hoc test, *p < 0.0*5* for comparison of treatments with the respective untreated controls

The induction of targets, such as GCL, requires the activation of upstream transcriptional co-activators. Several clustered genes identified by the WGCNA analysis are under the control of Nrf2. For an experimental insight into the dynamic intracellular stabilization and translocation of Nrf2 in live cells, Nrf2, heme oxygenase 1 (HMOX1), and sulfiredoxin (SRXN1) monoclonal HepG2-based BAC-GFP reporter lines (Wink et al. 2014, 2017, 2018) were exposed to NFT and to DEM (positive control) for maximal Nrf2 activation (Fig. 2a). Nrf2 translocation was assessed by quantification of the mean GFP intensity in the nucleus over time. For HMOX1 and SRXN1, global cellular GFP intensity was detected as an indicator of their respective expression levels. Nrf2-GFP reporter cells showed rapid Nrf2 translocation from the cytosol to the nucleus within minutes of DEM exposure (Fig. 2a). The high initial GFP intensity levels observed immediately after DEM exposure could be attributed to a delay of approximately 20 min between the addition of DEM and image acquisition (Fig. 2a). Compared with the rapid translocation of Nrf2 induced by DEM, NFT treatment resulted in a rather slow, but constant rise in the intensity of nuclear Nrf2 signals (Fig. 2a). A time lag was also observed with the downstream targets of Nrf2, HMOX1, and SRXN1, which exhibited plateaus between 12 and 24 h following activation with DEM, whereas NFT treatment resulted in a significantly less pronounced maximum with a delay of > 12 h compared with DEM (Fig. 2a). To exclude any effects associated with the procedure used for generation of a new monoclonal sub-clone, wild-type HepG2 cells were treated with varying concentrations of NFT or DEM for 24 h, fixed, and subjected to immunocytochemistry to visualize Nrf2 localization (Fig. 2b). Control cells exhibited an enrichment of Nrf2 protein in the vicinity of the nucleus. NFT or DEM treatment not only resulted in the translocation of a fraction of the Nrf2 into the nucleus but also showed dispersed localization in the cytosol. Quantification of the ratio of nuclear/cytosolic Nrf2 staining indicated a concentration-dependent increase in Nrf2 translocation from the cytosol to the nucleus for both NFT and DEM treatments (Fig. 2c), confirming the observations made with the Nrf2-GFP reporter line. These results indicate that NFT induced the activation and translocation of Nrf2 into the nucleus.

To further investigate NFT-dependent activation of Nrf2 and subsequent induction of GCL and glutathione synthesis, protein levels of the respective targets were investigated by Western blotting. In agreement with recent publications, Nrf2 displayed a biologically active molecular weight of > 100 kDa instead of the predicted 55–65 kDa band as expected (Lau et al. 2013). Both NFT and DEM induced a similar increase in total glutathione levels (Fig. 2d). Treatment of HepG2 cells with different concentrations of NFT or DEM for 24 h led to a concentration-dependent increase in Nrf2 and GCLC protein levels, whereas no significant change was observed in the levels of the modifier subunit, GCLM (Fig. 2e). These observations are consistent with the hypothesis that NFT activates Nrf2, leading to increased GCL synthesis, and subsequently, to a rise in intracellular glutathione levels.

### *Nrf2* knockdown prevents NFT-dependent increase in cellular glutathione levels

**Fig. 3:**
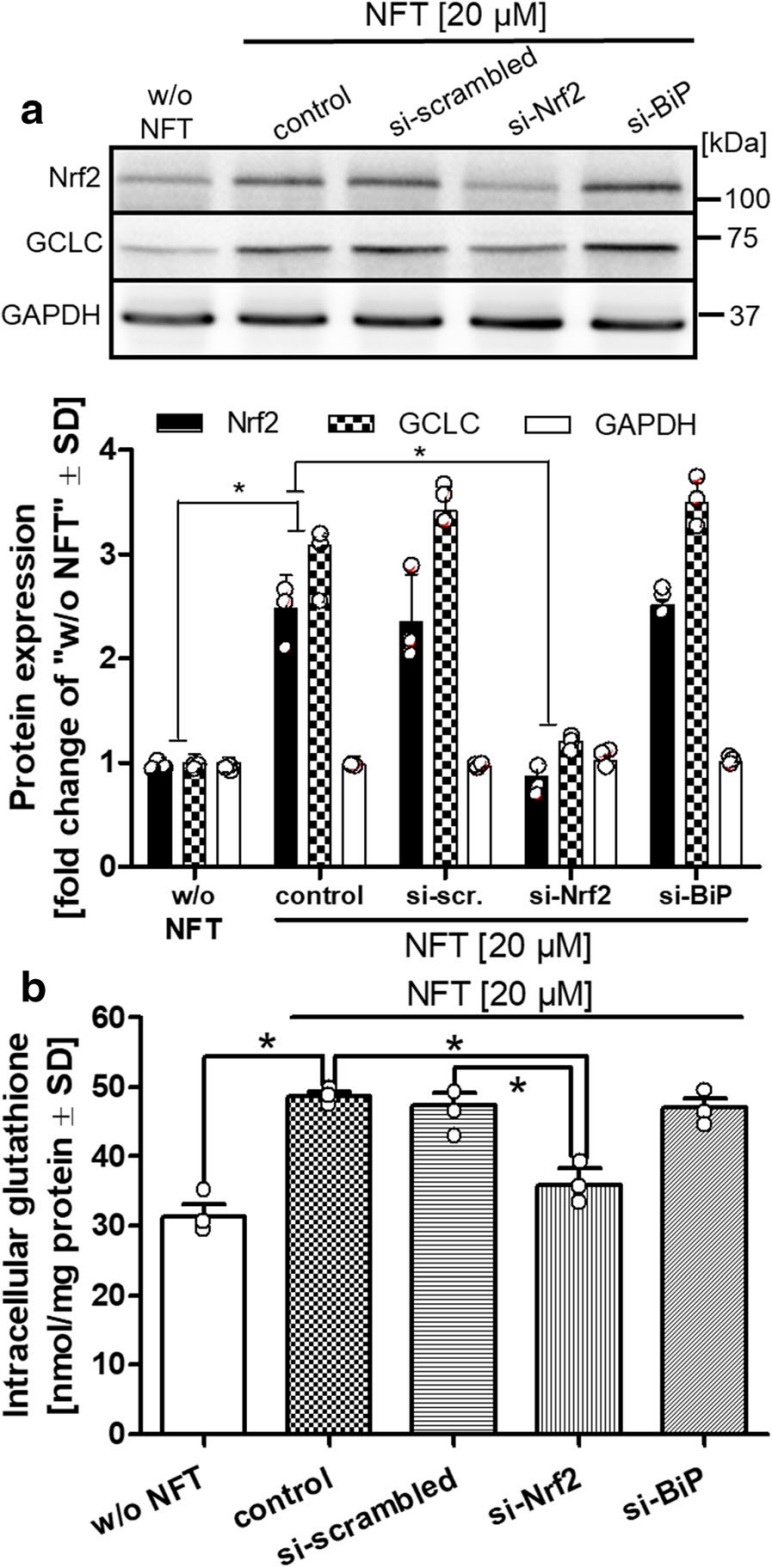
Nrf2 knockdown prevents NFT-dependent elevation of glutathione levels. **a** Nrf2, BiP (GRP78), or random targets (scrambled) were silenced in HepG2 cells by utilizing 40 μl of a 10 μM siRNA stock solution per 10^6^ cells for 24 h. Cells were then exposed to NFT (20 μM) for 48 h, and cell homogenates were analyzed by western blotting for the levels of Nrf2, GCLC, and GAPDH. For quantification, band intensities were normalized to those of GAPDH. Values of untreated control cells (w/o NFT) were defined as unity, and all other bands were expressed as fold change compared to control. **b** The siRNA-silenced cells were analyzed for their intracellular content of glutathione. Differences were tested for significance by one-way ANOVA, followed by Bonferroni’s post hoc test, *p < 0.05 for comparison of NFT treatment vs. untreated controls and for comparison of NFT treatment vs. siRNA knockdown. Data are means ± SD of three independent experiments. Individual values of the 3 biological replicates are indicated by dots

To examine the correlation between Nrf2 activation and the increase in glutathione levels after NFT treatment, Nrf2 protein levels were experimentally lowered by siRNA-mediated knockdown, which was confirmed by Western blotting (Fig. 3a). Binding immunoglobulin protein (BiP/Grp78), an endoplasmic reticulum chaperon, was selected as a negative control. Both scrambled and BiP/Grp78 siRNAs had no influence on Nrf2 protein levels. Importantly, protein levels of GCLC followed the trend observed with Nrf2 protein: GCLC levels were elevated by NFT treatment, and anti Nrf2-siRNA largely prevented this increase (Fig. 3a). Intracellular glutathione also followed the trend of GCLC, exhibiting an NFT-dependent increase that was reversed by anti Nrf2-siRNA to levels comparable to those observed in untreated HepG2 cells (Fig. 3b). These findings indicate that NFT treatment activated Nrf2, which caused the rise in GCL and glutathione protein levels. Next, we investigated the mechanism by which NFT triggered the activation of Nrf2 at the molecular level.

### Formation of the NFT radical anion by cellular reductases

**Fig. 4:**
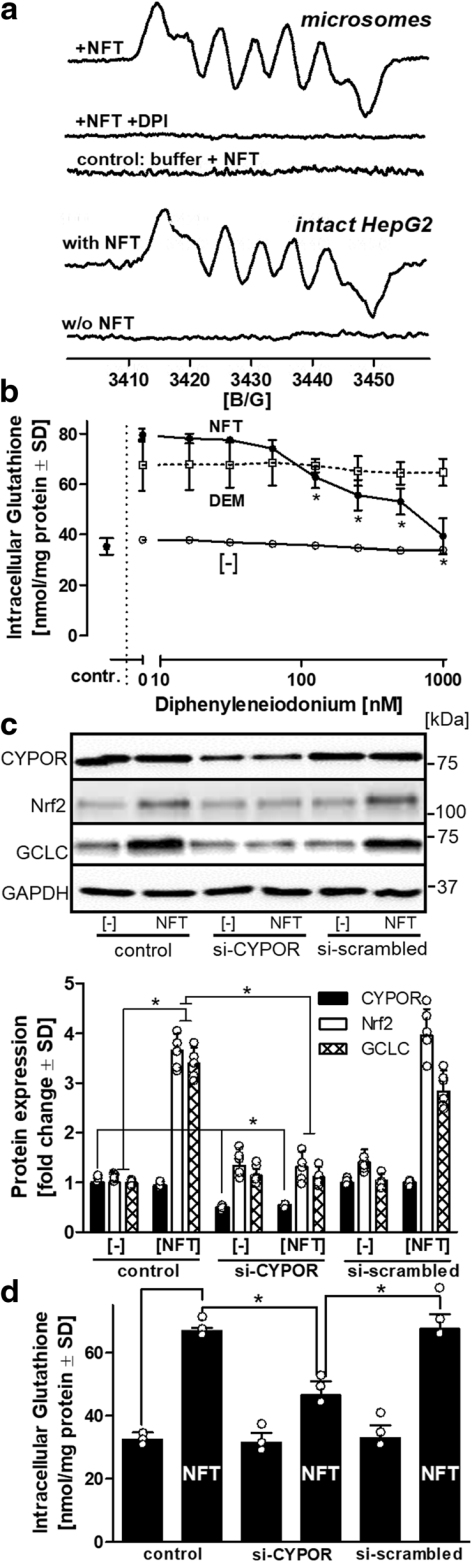
Activation of NFT. **a** EPR spectroscopic detection of the NFT radical anion. Microsomes (1 mg/mL protein) prepared from HepG2 in potassium phosphate buffer, pH 7.4, were supplemented with NFT (5 mM) and NADPH (10 mM) in the presence or absence of the flavoenzyme inhibitor, diphenyleneiodonium (DPI) (10 μM). Spectra were background-corrected by subtraction of the recordings obtained in absence of NFT. A total of 117 scans were recorded for microsomal preparations. Intact detached HepG2 cells (10^7^ cells/mL) in serum-free medium were incubated in the absence or presence of NFT (5 mM). The spectrum obtained with intact HepG2 cells represents the integration of spectra from 12 scans. **b** HepG2 cells were exposed to DPI for 1 h, followed by the addition of NFT (40 μM) or diethyl maleate (DEM) (100 μM) for an additional 24 h. As reference, cells were exposed only to indicated concentrations of DPI. Control cells received no DPI, NFT, or DEM. Intracellular levels of glutathione were determined. **c** Knockdown of cytochrome P450 reductase (*CYPOR*). HepG2 cells were treated with siRNA (80 μL of a 10 μM stock solution per 2 × 10^5^ cells) targeting CYPOR for 48 h. Scrambled siRNA was applied as negative control. The cells were then incubated for an additional 24 h in the presence or absence of nitrofurantoin (NFT) (40 μM). Proteins were separated by gel electrophoresis and GAPDH levels used for normalization. For each individual target, protein levels in untreated control cells were defined as unity, and alterations are expressed as fold changes compared to untreated controls. Data are means of 4 independent experiments ± SD. Individual values are indicated by dots. **d** In parallel, intracellular glutathione content was assessed under identical experimental conditions. Data are means of 3 independent experiments ± SD. Individual values are indicated by dots. Differences (**b***,* **d**) were tested for significance by one-way ANOVA, followed by Bonferroni’s post hoc test, *p < 0.05 for (**b**): comparison of treatments NFT or DEM with [−] cells; (**c**) + (**d**): comparison of NFT vs. untreated control and NFT vs. siRNA knockdowns

To elucidate the mechanism of Nrf2 activation by NFT, we focused on the ability of NFT to yield an activated species capable of oxidizing critical sulfhydryl groups in the Nrf2-Keap1 complex. The formation of an NFT radical anion has been demonstrated in enzymatic assays following a one-electron reduction, e.g., by xanthine oxidase (Miller et al. 2002). HepG2-derived microsomes and EPR spectroscopy allowed us to confirm the formation of the NFT radical anion (Fig. 4a). This is the first study to demonstrate the formation of the NFT radical anion by EPR spectroscopy in live HepG2 cells treated with NFT (Fig. 4a). Diphenyleneiodonium (DPI), a flavoenzyme inhibitor, not only prevented the formation of the NFT radical anion in microsomal preparations (Fig. 4a) but also inhibited the NFT-dependent increase in intracellular glutathione levels in a concentration-dependent manner (Fig. 4b). However, it had no effect on DEM-induced glutathione synthesis. These observations indicate the involvement of cellular reductases in the activation of NFT and exclude the possibility of autoxidation based on EPR measurements with NFT in the absence of microsomes or cells. Because cytochrome P450 reductase (CYPOR) is highly expressed in HepG2 cells and in primary hepatocytes (Schulz et al. 2019), we investigated the effects of its siRNA-based knockdown on glutathione levels. Western blotting revealed that knocking down *CYPOR* prevented Nrf2 accumulation and GCLC induction upon NFT treatment (Fig. 4c). The reduction in GCLC protein levels led to reduced NFT-dependent stimulation of glutathione synthesis (Fig. 4d). These observations suggest a significant contribution of CYPOR to NFT-induced Nrf2 activation in the HepG2 model, but also indicate that other cellular flavoprotein reductases are likely to be involved in the formation of the NFT radical anion and its subsequent activation of Nrf2 and synthesis of GCLC and glutathione.

### Duration of NFT-dependent increase in glutathione levels

**Fig. 5:**
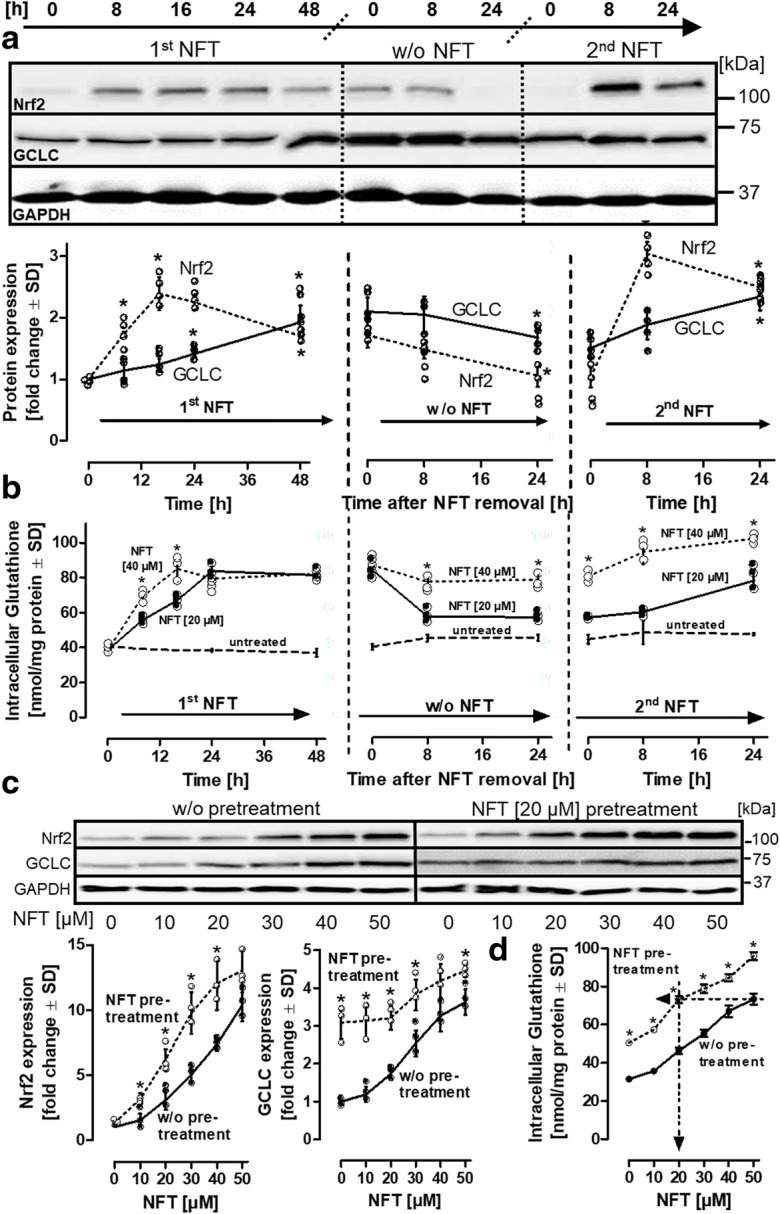
Repeated NFT dosing. **a** HepG2 cells were treated with 20 μM of NFT for different time intervals for up to 48 h (= 1^st^ NFT). Medium was then changed, and the cells were maintained in the absence of NFT for additional 24 h (= w/o NFT). After this period, NFT was re-added, and cells were incubated for up to one day (= 2^nd^ NFT). Medium changes are indicated by the dotted separation lines. Samples were adjusted for equal protein content; western blots were stained for Nrf2 and GCLC. For Western blot quantification, the untreated control bands (*t* = 0) were normalized to unity, and band intensities were indicated as fold changes related to the respective controls. **b** Cells were treated with NFT (20 μM or 40 μM) and analyzed for their intracellular levels of glutathione. **c** HepG2 cells were treated with or without NFT (20 μM) for 48. The cells were then maintained in the absence of NFT for 24 h, followed by the addition of varying concentrations of NFT as indicated for a period of 24 h. Stabilization of Nrf2 and induction of GCLC were analyzed by western blot analysis. **d** In parallel, intracellular levels of glutathione were determined. The arrows highlight the maximal elevation of intracellular glutathione content, induced by an initial treatment with NFT in comparison to cells that were exposed to NFT pretreatment. Data are means of 4 (**a**), or 3 (**b** + **c**) independent experiments ± SD. Individual values of biological replicates are indicated by dots. In (**a**), differences were tested for significance by one-way ANOVA, followed by Bonferroni’s post hoc test, *p < 0.05. In (**b**) and (**c**), differences were tested for significance by two-way ANOVA (NFT 20 μM vs. 40 μM for individual time intervals), followed by a Bonferroni’s post hoc test *p < 0.05

To determine the duration and the response of glutathione following withdrawal and re-addition of NFT, HepG2 cells were treated with NFT (20 μM) for 48 h, followed by withdrawal of NFT for 24 h and a subsequent re-addition of NFT for an additional period of 24 h (Fig. 5a). Treatment with NFT resulted in an accumulation of Nrf2 protein after 8 h, and an induction of GCLC synthesis beyond 24 h. Following withdrawal of NFT, Nrf2 levels rapidly declined below the detection limit, whereas GCLC levels remained significantly higher than those observed in the untreated control even after 24 h in the absence of NFT. The same experiment was performed with a higher concentration of NFT (40 μM) (Suppl. Fig. 8) and revealed a higher nuclear accumulation of Nrf2 protein and faster induction of GCLC synthesis than that observed with 20 μM NFT (Suppl. Fig. 8). Re-addition of NFT resulted in Nrf2 re-accumulation, GCLC and glutathione synthesis followed this trend in cells treated with 20 μM NFT. Cells that were initially exposed to 40 μM NFT also exhibited a second wave of Nrf2 accumulation (Suppl. Fig. 8) as well as GCLC and glutathione synthesis (Fig. 5b). In the 40 μM pretreatment group, levels remained high even in the absence of NFT and showed only a modest increase in response to the re-addition of NFT (Fig. 5a, b) (Suppl. Fig. 8).

Standard oral doses of NFT have been found to result in plasma NFT levels in the low micromolar range (Novelli and Rosi 2017; Wijma et al. 2019). To determine the amount of NFT required after its initial addition and a washout phase to maintain elevated glutathione levels, cells were pretreated with 20 μM NFT for 48 h, maintained in NFT-free medium for 24 h, and subsequently treated with varying concentrations of NFT over 24 h. Consistent with observations in Fig. 5a, Nrf2 accumulation was more pronounced in the NFT pretreatment setup (Fig. 5c). Moreover, pretreated cells displayed higher basal GCLC levels than cells without pretreatment, and the second NFT treatment led to a relatively modest additional increase (Fig. 5c). Importantly, pretreatment with 20 μM NFT reduced the requirement for NFT after a washout phase to achieve the same intracellular glutathione levels (Fig. 5d). These results indicate that (a) treatment with a high initial concentration (40 μM) of NFT led to sustained elevation of intracellular glutathione levels for up to 48 h; (b) consecutive treatments with NFT (including intermittent washout phases) exhibited an additive effect with respect to the increase in glutathione levels. Even after three cycles of NFT exposure (with 24-h washout phases), no signs of desensitization were observed (data not shown). In conclusion, elevated glutathione levels can be maintained by low micromolar concentrations of NFT in the range that can be expected in organs, such as the liver, over extended periods of time. 

### NFT pretreatment protects against various stress insults

**Fig. 6:**
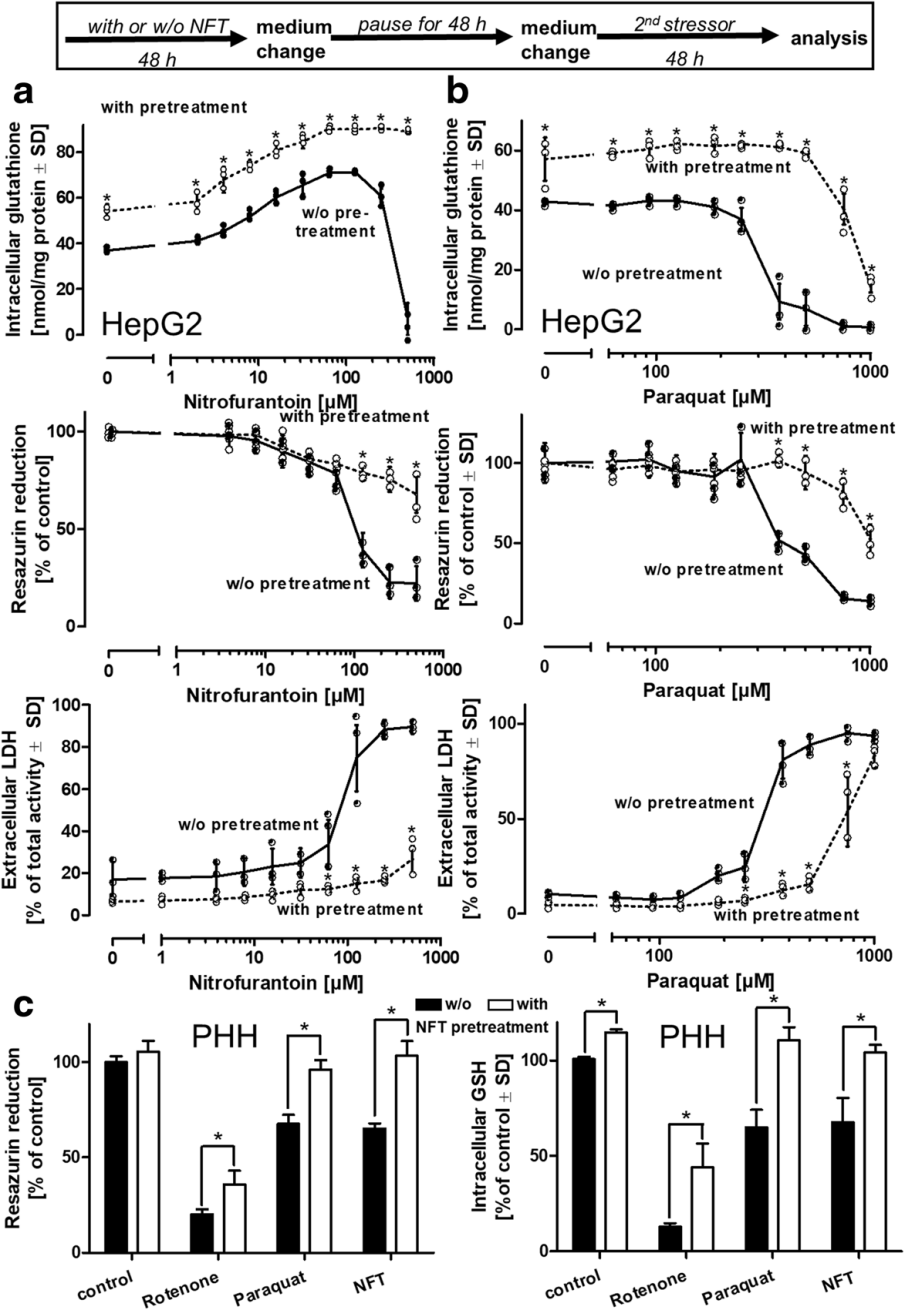
NFT pretreatment protects from various insults. HepG2 cells were pretreated with NFT (40 μM) for 48 h, followed by a medium change and maintenance in the absence of NFT for an additional 48 h. Then, **a** NFT or **b** the redox cycler, paraquat, were added at a toxic concentration range for 48 h. Cell viability was analyzed by reduction of resazurin and by the LDH release assays, and cell homogenates were analyzed for their glutathione content. Data are means of 3 independent experiments ± SD, individual data are illustrated by dots. **c** Primary human hepatocytes (PHH, 150,000 cells/cm^2^) from three individual donors (HUM4229, HUM4108, HUM181501B) were maintained in the presence of NFT (25 μM) for 48 h, followed by a period of 48 h in the absence of NFT. Then, rotenone (5 μM), paraquat (500 μM), or a damaging concentration of NFT (125 μM) were added for 24 h. Viability was assessed by the resazurin reduction assay, cell homogenates were assessed for their glutathione content. Differences were tested for significance by two-way ANOVA (pretreatment vs. w/o pretreatment for individual NFT or paraquat concentrations), followed by a Bonferroni’s post hoc test *p < 0.05

To investigate the influence of elevated glutathione levels on the resilience of cells against various stress insults, HepG2 cells were pretreated for 48 h with 40 μM NFT, and the cells were washed and maintained for 48 h in the absence of NFT. An additional medium change was then accompanied by the addition of toxic concentrations of either NFT (Fig. 6a) or the redox cycler, paraquat (Fig. 6b). NFT pretreatment resulted in a marked shift of LD_50_ toward higher concentrations of either NFT or paraquat required for the induction of cell damage compared to cells without pretreatment. These observations were also made with mechanistically different secondary stressors such as the mitochondrial complex I inhibitor, rotenone, or proteasomal inhibitors such as MG-132 or bortezomib (Suppl. Fig. 9). In order to verify the protective influence of NFT in a model that closer resembles the situation in vivo, PHH from three individual donors were employed (Fig. 6c). PHH that were exposed to NFT before displayed less damage evoked by stressors such as paraquat or rotenone and hence fully confirmed the observations made with the HepG2 model. Thus, the results of this study demonstrate the potential role of NFT as a tool for targeted stimulation of glutathione synthesis to enhance cellular resistance toward various adverse conditions.

## Discussion

### Activation of antioxidant responses by Nrf2

The transcription factor, Nrf2, has emerged as a promising target for selective pharmacological stimulation of protective cellular pathways (Robledinos-Antón et al. 2019; Satoh and Lipton 2017; Abed et al. 2015). Activation of Nrf2 and a subsequent induction of GCL have been reported for various oxidants and electrophiles such as DEM, H_2_O_2_, quinones, 4-hydroxynonenal, methyl mercury, arsenicals, and plasmonic gold nanoparticles (Zhang et al. 2007; Sekhar et al. 1997; Woods and Ellis 1995; Iles and Liu 2005; Schuliga et al. 2002; Krzywanski et al. 2004; Goldstein et al. 2016; Yoshida et al. 2014). However, these compounds are associated with Nrf-2-independent adverse effects, which prevented their clinical use despite their beneficial induction of glutathione synthesis.

The antibiotic, NFT, is primarily used for the treatment of uncomplicated urinary tract infections (Hummers-Pradier and Kochen 2002). Although approximately 1 in 1500 patients exhibit signs of chronic liver injury in long-term prophylactic therapy, acute liver injury due to NFT is quite rare, with approximately 0.3 cases per 100,000 prescriptions (LiverTox 2020). Peak plasma concentrations were reported to be in the range of 1–15 μM (Novelli and Rosi 2017; Wijma et al. 2019). To our knowledge, tissue NFT concentrations have not yet been determined in specific human organs. The cell culture experiments in this study indicate that NFT concentrations in the range of 5–50 μM were optimal for the induction of de novo glutathione synthesis. The NFT concentrations observed in the in vitro experiments for glutathione stimulation in this study can hence be considered to be representative or at least close of the concentrations that could be expected in vivo. Stimulation of de novo glutathione synthesis by NFT concentrations in this range was observed in HepG2 cells, PHHs, or human neuronal cells, but not to the same extent in other cell types such as human iPSC-derived astrocytes. These findings indicate that although the observed protective effects are not limited to hepatocytes, they require defined cell-specific components for NFT activation. Hepatoma cell lines, such as HepG2, in general display lower expression levels of hepatic phase I and phase II enzymes compared with primary hepatocytes, thus questioning their informative value for the situation in vivo. Despite several constraints, PHH rank among the most representative models for human hepatocytes. In the present study, PHH not only indicated an upregulation of glutathione upon NFT expose but also confirmed an elevated resilience of NFT pre-treated cells toward other stressors. Furthermore, changes in gene expression patterns of NFT-exposed PHH or HepG2 indicated (depending on the NFT concentrations applied) correlation coefficients of > 0.5. These observations qualified the HepG2 model to study the mechanisms described herein.

### Formation of the NFT radical anion is required for Nrf2 activation

As activation of Nrf2 requires the oxidation of critical sulfhydryl groups in its interaction partner, Keap1, we investigated the mechanism of this activation by NFT. Incubation of NFT with cell or tissue homogenates, microsomal preparations, or isolated mitochondria revealed one-electron reduction of NFT to yield an NFT radical anion (^•^NFT^−^) by flavoprotein-reductases such as xanthine oxidase, aldehyde dehydrogenase, thioredoxin reductase, cytochrome b_5_ reductase, and cytochrome P450 reductase (CYPOR) (Miller et al. 2002; Moreno et al. 1984; Letelier et al. 2004; Szilagyi et al. 2018; Minchin et al. 1986). Subsequent redox cycling of the NFT radical generates superoxide (O_2_^• −^) and O_2_^• −^-derived reactive oxygen species (Wardman 1985; Sasame and Boyd 1979). In intact HepG2 cells, the pan-flavoenzyme inhibitor, DPI, prevented the NFT-stimulated increase in glutathione levels, indicating the involvement of one or more of these enzymes in NFT-dependent activation of Nrf2 (Fig. 4b). Thus, this is the first study to demonstrate the formation of ^•^NFT^−^ in both enzyme preparations and intact cells. The formation of ^•^NFT^−^ in an aerobic intracellular environment raises the question of its stability and its interaction with biological molecules such as the omnipresent glutathione. In the past, there has been controversy regarding the interaction of ^•^NFT^−^ with glutathione (Silva et al. 1993; Núñez-Vergara et al. 2000). In a series of EPR experiments, Miller et al. clearly showed that in the presence of millimolar GSH and 40 μM O_2_ (as in normal tissue), the interaction of ^•^NFT^−^ with O_2_ would outcompete its reaction with GSH by a factor of 10,000 (Miller et al. 2002). Even in the presence of only 1 μM O_2_—representative of hypoxia or oxygen-deprived areas of the liver under homeostatic conditions—the interaction of ^•^NFT^−^ with O_2_ is still 200-fold faster than its interaction with GSH (Miller et al. 2002). The reduction of O_2_ by ^•^NFT^−^ yields NFT and superoxide (^•^O_2_^−^). As superoxide is subject to efficient dismutation by cellular superoxide dismutases, it is possible that ^•^NFT^−^ might directly interact with Keap1 to induce the observed release of Nrf2 and its escape from proteasomal degradation. Our observations of a concentration-dependent inhibition of NFT-dependent glutathione synthesis stimulation (Fig. 4b) by DPI and the absence of such inhibition by antioxidants such as ascorbic acid, the peroxynitrite-scavenger uric acid, ebselen, and even the spin-trap TEMPONE-H (Suppl. Fig. 10) indicate a complex interaction between NFT derivatives and the Nrf2 pathway. The identity of NFT derivatives and/or reactive oxygen species interacting with the Nrf2-Keap1 complex were not identified in the course of the present work, but will be investigated in future studies.

Reports in the literature provide experimental evidence for one-electron reduction of NFT by recombinant CYPOR (Wang et al. 2008). Because HepG2 cells possess higher CYPOR activity than primary hepatocytes (Schulz et al. 2019), we selected CYPOR as a target for knockdown. A reduction in CYPOR protein levels prevented NFT-dependent accumulation of Nrf2 protein and partially attenuated the NFT-dependent increase in glutathione levels. However, it should be mentioned that, for technical reasons, siRNA-based knockdown of CYPOR was never complete. These findings underline the important role of CYPOR as an electron donor for the reduction of NFT, but do not exclude the involvement of other reductases in NFT reduction.

### NFT-dependent activation of the Nrf2 pathway elevates cellular resilience

Nrf2/Keap1 ranks among the most relevant cellular signaling elements that combine sensing of oxidative stress with transcriptional activation of downstream targets involved in antioxidant defense. DEM has been described as an excellent activator of the Nrf2 pathway in the HepG2 model (Bischoff et al. 2019) and was therefore used as a positive control. Other recently suggested Nrf2 activators, such as the cell-permeable alkylating agent, dimethyl itaconate (Mills et al. 2018), sulforaphane (Thimmulappa et al. 2002), or *tert*-butyl hydroquinone (Krzywanski et al. 2004) (for the induction of lipid peroxidation to yield, e.g., 4-hydroxynonenal or malondialdehyde) were tested, but resulted in only a modest increase in cellular glutathione levels (Suppl. Fig. 11). These observations underline the potency of NFT as a pharmacological Nrf2 activator. Although the time course of Nrf2 stabilization and nuclear translocation indicated a rapid increase with a peak at approximately 6 h for DEM, and a slow but gradual rise triggered by NFT, maximal accumulation of Nrf2 protein, as well as the induction of GCLC and glutathione synthesis, were almost identical for both NFT and DEM after incubation periods of and beyond 24 h (Fig. 2). Based on the published literature on Nrf2, the rise of Nrf2 protein levels is likely due to impaired degradation of the Nrf2 protein, rather than an induction of its transcription or translation. This speculation is supported by the evidence that Nrf2 mRNA levels were not significantly influenced by NFT treatment (not shown). The constant levels of marker proteins such as GAPDH, combined with observations indicating that knockdown of the UPR element, *BiP/Grp78*, revealed no detectable effects on Nrf2, GCLC, or glutathione, further support the conclusion that NFT-mediated stabilization of Nrf2 is a major contributor to its accumulation and the subsequent stimulation of GCL and glutathione synthesis.

In the context of repeated NFT exposure, Nrf2 displayed a more pronounced accumulation in response to a second NFT treatment. These observations are in contrast to previous findings in the same Nrf2-GFP-HepG2 reporter model exposed to DEM or *tert*-butylhydroquinone, which indicate a lower Nrf2 response toward repeated treatments (Bischoff et al. 2019). DEM and *tert*-butyl-hydroquinone activate Nrf2 either directly or via the formation of lipid hydroperoxides. In contrast to NFT, they required no activation by cellular reductases (Fig. 4b). Hence, the observed differences in Nrf2 activation might be a consequence of different modes of activation and deserve consideration during long-term use of NFT. Interestingly, repeated NFT treatment resulted in higher GCLC levels in the days after removal of the compound and an elevated induction in response to a second exposure to NFT, similar to the previously published pattern of sulfiredoxin synthesis upon repeated exposure to DEM or *tert*-butylhydroquinone (Bischoff et al. 2019). This comparison clearly highlights an adaptation of the cells toward a more sensitized state upon initial NFT exposure, thus enabling a pharmacological elevation of glutathione levels by even lower doses of repeated NFT treatments.

One of the most intriguing observations in this context was the desensitization of cells pretreated with NFT for glutathione induction in response to secondary stressors. Elevated glutathione levels protected HepG2 cells not only from oxidative stress (generated by the redox cycler, paraquat) but also from mechanistically diverse stressors such as the inhibition of mitochondrial complex I by rotenone or the inhibition of the proteasomal system by MG-132 and bortezomib (Suppl. Fig. 9). NFT-dependent elevation of glutathione levels even protected the cells from subsequent treatment with toxic NFT concentrations. These in vitro observations not only provide an initial basis for in vivo treatment regimens focusing on the stimulation of endogenous hepatic glutathione levels, but could also contribute to an optimization of treatment intervals to limit the risk of adverse effects when NFT is administered as an antibiotic.

## Conclusions

Nrf2 is a master regulator of several protective stress response pathways and it is not surprising that it became a highly competitive target for pharmacological modulation (Cuadrado et al. 2018). The clinical value of most of the so far described Nrf2 activators however was rather limited, mostly due to undesired interactions of these electrophiles with other cellular targets. To limit the risk of withdrawal during clinical studies, we pursued the strategy of drug repurposing to benefit from already established safety records. NFT stands out among other Nrf2 activators as it (i) allows activation of Nrf2 and stimulation of glutathione synthesis at concentrations (low micromolar range) reported in the plasma of patients receiving standard oral doses of the antibiotic; and (ii) induces a quantitative rise of Nrf2 and glutathione that could only be achieved by the highest non-toxic concentrations of the potent experimental Nrf2-activator DEM. These observations indicate that NFT could be a promising candidate for a transient and targeted treatment of conditions associated with oxidative stress.

## Supplementary information

ESM 1 (DOCX 1210 kb)

## Data Availability

The authors declare that all data presented are publicly available upon request.
