# Supplementary material for: Stimulation of de novo glutathione synthesis by nitrofurantoin for enhanced resilience of hepatocytes
Source: Cell Biol Toxicol. 2021 May 22;38(5):847–64. doi: 10.1007/s10565-021-09610-3 (PMC9525367; doi:10.1007/s10565-021-09610-3)
Supplement: Supplementary file 1 — (DOCX 1210 kb) [file 10565_2021_9610_MOESM1_ESM.docx]

**Supplementary Figures**

**Stimulation of *de novo* glutathione synthesis by nitrofurantoin for enhanced resilience of hepatocytes**

Lukas Wijaya, Carina Rau, Theresa Braun, Serif Marangoz, Vincent Spegg, Matthijs Vlasveld, Wiebke Albrecht, Tim Brecklinghaus, Hennicke Kamp, Joost B. Beltman, Jan Hengstler, Bob van de Water, Marcel Leist, Stefan Schildknecht

| **Compounds** | **c_max_ plasma** | **Oxidative stress**  **(SRXN1-GFP)** | **Oxidative stress**  **(HMOX1-GFP)** | **Glutathione**  **(>50% increase)** |
| --- | --- | --- | --- | --- |
| **Amiodarone** | **0.8** | **65** | **32** | **-** |
| **Azathioprine** | **0.3** | **14** | **7** | **-** |
| **Ciprofloxacin** | **6.6** | **132** | **66** | **-** |
| **Diclofenac** | **10.1** | **202** | **101** | **375** |
| **Ketoconazole** | **6.6** | **33** | **66** | **-** |
| **Nitrofurantoin** | **6** | **30** | **30** | **6** |
| **Paracetamol** | **140** | **5558** | **4168** | **2750** |
| **Phenytoin** | **22** | **869** | **869** | **125** |
| **Tolcapone** | **22** | **220** | **220** | **-** |
| **Troglitazone** | **6.4** | **64** | **254** | **50** |
| **Valproic acid** | **242.2** | **4855** | **4855** | **6250** |

**Suppl. Fig. 1 Compound screening.** HepG2 reporter cell lines, expressing tagged SRXN1 or HMOX1 were exposed to the compounds listed. Maximal human plasma concentrations (c_max_  in µM) described in the literature were applied as reference points. The table displays the induction of SRXN1 or HMOX1 as downstream elements of the oxidative stress response pathway. In addition, intracellular glutathione was detected. The table illustrates the compounds that initiated an induction of the reporters and/or an induction of glutathione synthesis. Elevation of glutathione was defined as the increase of intracellular glutathione by 50 % or higher. The antibiotic nitrofurantoin exhibited an induction of SRXN1 and HMOX1, as well as an increase in glutathione at concentrations lower than 10 x c_max_ and was therefore investigated in detail (concentrations in µM).


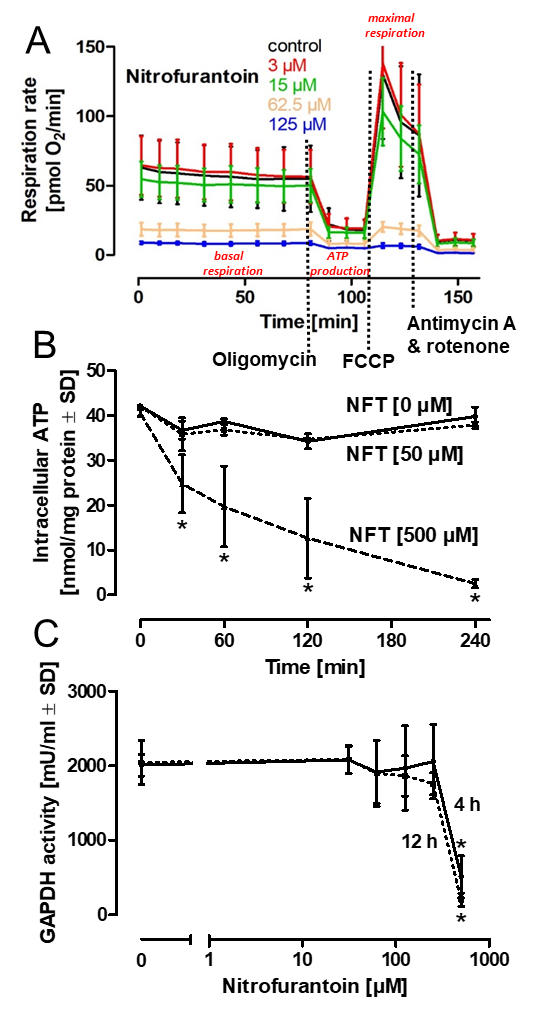


**Suppl. Fig. 2. Inhibition of cellular energy production. *A)*** Oxygen consumption rate of a Seahorse Mito Stress Profile. HepG2 were seeded at a 24 well Seahorse plate at a density of 50.000 cells/well. After two days, medium was changed and nitrofurantoin (NFT) was added at the indicated concentrations for 1 h. Following standard protocols, the ATP synthase inhibitor oligomycin was added to inhibit electron flow and oxygen reduction by stimulation of maximal inner mitochondrial transmembrane proton gradient buildup. For collapse of the proton gradient and consequently maximal electron flow along the mitochondrial respiratory chain, the uncoupler FCCP was added, followed by the addition of a combination of complex I inhibitor rotenone and complex III inhibitor antimycin A for the inhibition of the respiratory chain and oxygen reduction. ***B)*** Acute treatment of HepG2 with NFT. Cells were treated for the indicated time intervals with an intermediate (50 µM) and a high concentration (500 µM) of NFT. Intracellular ATP was detected at the respective time intervals as indicated. ***C)*** Intact HepG2 were treated with NFT (500 µM) as indicated for 4 h or 12 h. Cells were homogenized, and the cytosolic fraction was assessed for GAPDH enzyme activity.


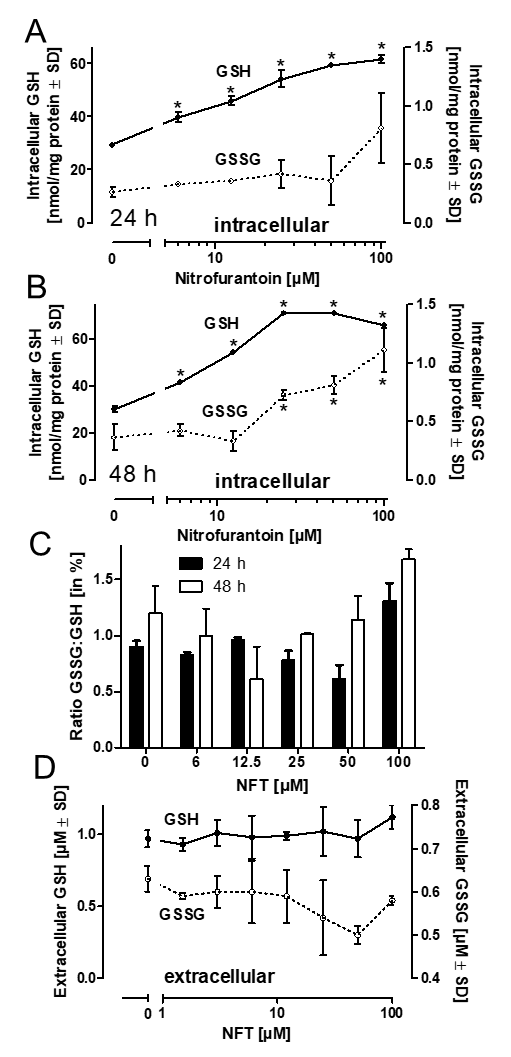


**Suppl. Fig. 3. Detection of reduced (GSH) and oxidized (GSSG) glutathione.** HepG2 were treated with NFT as indicated for 24 h (***A***) or 48 h (***B***), and the intracellular amounts of GSH and GSSG were detected. ***C)*** Ratio between GSSG:GSH expressed as percentage. ***D)*** Detection of GSH and GSSG in the supernatant of HepG2 cells exposed to NFT as indicated for 24 h.

**Suppl. Fig. 4.** Intracellular glutathione in primary human hepatocytes (PHH) and HepG2. Cells were seeded and allowed to rest for 2 days. Due to the lack of significant proliferation of PHH and continuous proliferation of HepG2, cells were then detached, counted, and 50.000 cells (PHH or HepG2) were assessed for intracellular glutathione.


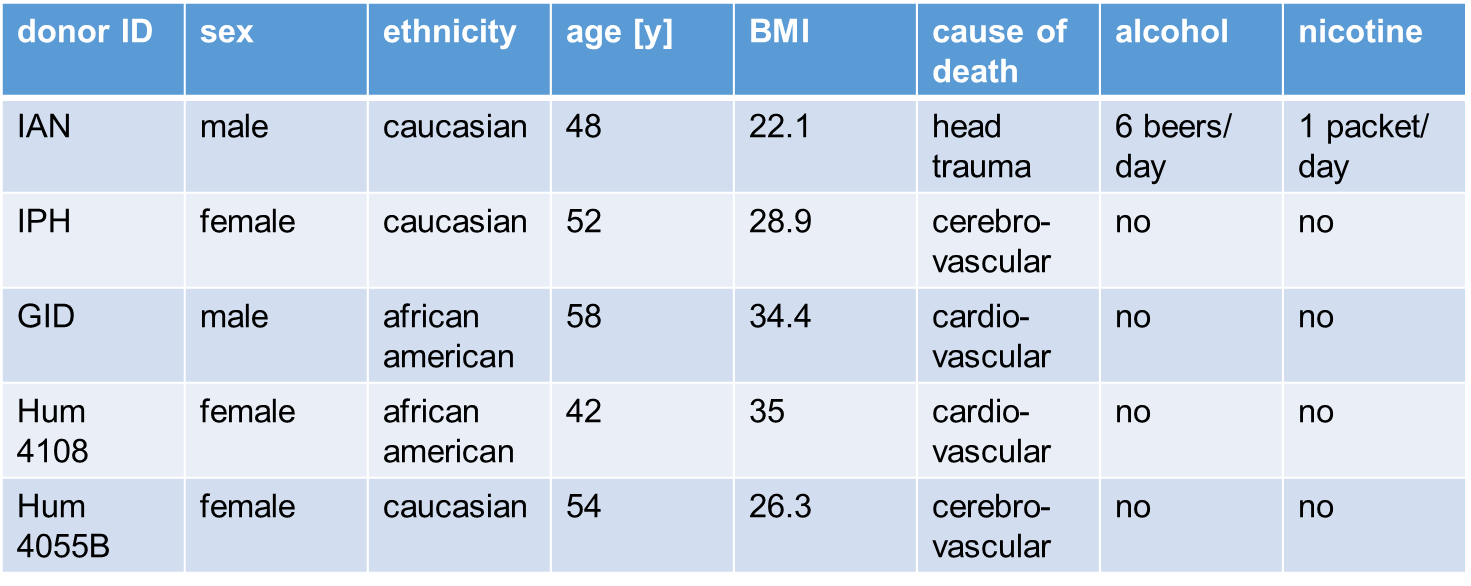


**Suppl. Fig. 5. Primary human hepatocyte background information.**

**
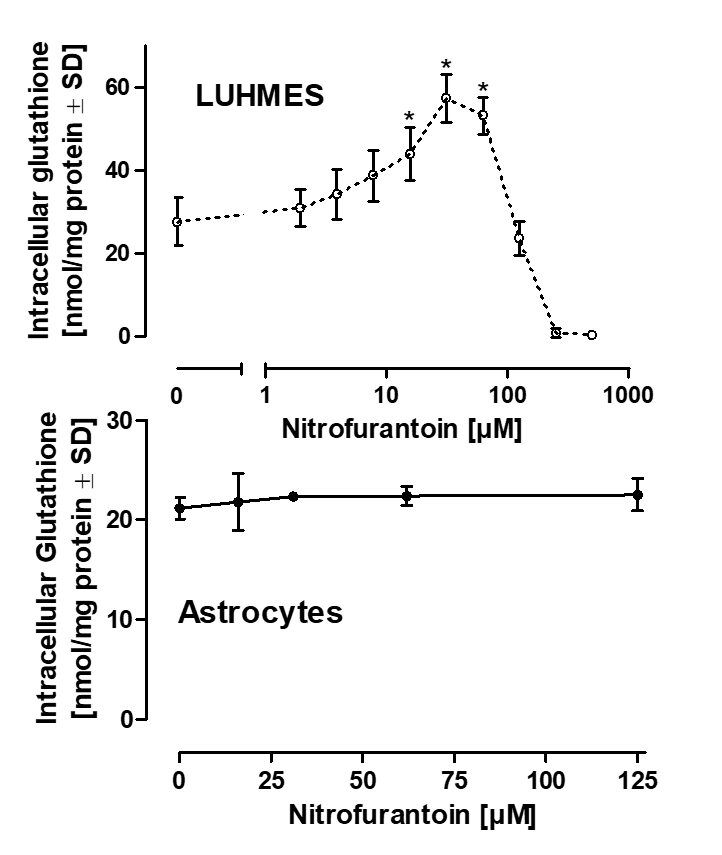
**

**Suppl. Fig. 6. Stimulation of glutathione synthesis by NFT in other cell types.** As an alternative to hepatocytes, the human neuronal cell line LUHMES, or iPSC-derived human astrocytes, were employed. The cells were exposed to NFT for 48 h. Astrocyte differentiation according to: Brüll et al. ALTEX, 2020; 37(3), 409-428

A

B

**Suppl. Fig. 7. Pearson correlation analysis of gene networks (modules) in HepG2 and PHH exposed to NFT.** (a) In the correlation matrix, the highest Person correlation values between HepG2 and PHH are indicated in blue (-1). (b) Correlation plots with the highest correlation coefficients between HepG2 and PHH. Horizontal axes: PHH treated with NFT (125 µM) for 24 h. Vertical axes: 1^st^ plot: HepG2 treated with NFT (120 µM) for 24 h; 2^nd^ plot: HepG2 treated with NFT (30 µM) for 24 h; 3^rd^ plot: HepG2 treated with NFT (60 µM) for 24 h.

**
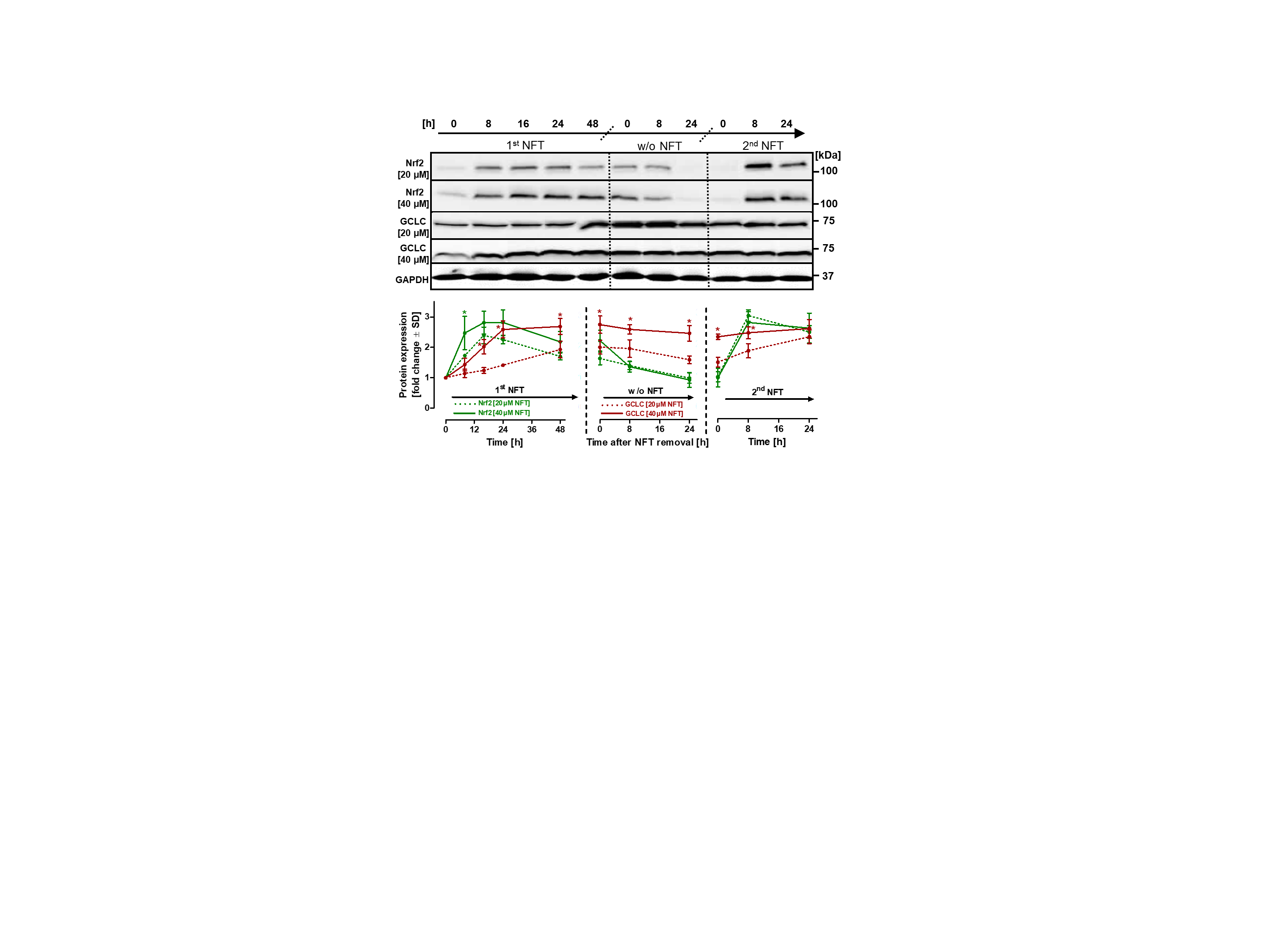
**

**Suppl. Fig. 8. Repeated NFT dosing.** ***A)*** HepG2 were treated with either 20 µM or 40 µM of NFT for different time intervals for up to 48 h (= 1^st^ NFT). Medium was then changed, and the cells were maintained in the absence of NFT for additional 24 h (= w/o NFT). After this period, NFT was re-added and cells were incubated for up to one more day (=2^nd^ NFT). Medium changes are indicated by the dotted separation lines. Samples were adjusted for equal protein content, Western blots were stained for Nrf2 and GCLC. For Western blot quantification, the untreated control bands (t = 0) were normalized to unity, and band intensities are indicated as fold changes related to the respective controls. Differences were tested for significance by two-way ANOVA (NFT 20 µM vs. 40 µM for individual time intervals), followed by a Bonferroni’s post hoc test * < 0.05.


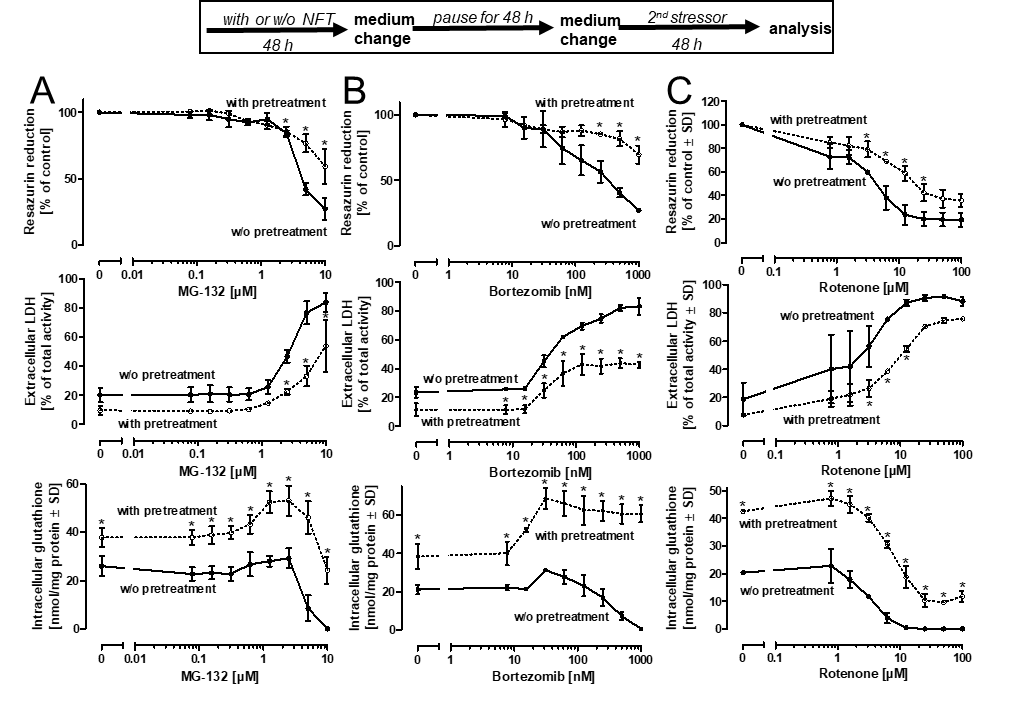


**Suppl. Fig. 9. NFT pre-treatment protects from various insults.** HepG2 were pre-treated with NFT (40 µM) for 48 h, followed by a medium change and an incubation period for 48 h in the absence of NFT. Then, the proteasome inhibitors MG-132 (***A***) or bortezomib (***B***), respectively the mitochondrial complex I inhibitor rotenone (***C***) were added as second stressor for 48 h. Cell viability was analyzed by the resazurin reduction and by the LDH release assays, and cell homogenates were analyzed for their glutathione content. Differences were tested for significance by two-way ANOVA (pretreatment vs. w/o pretreatment), followed by a Bonferroni’s post hoc test * < 0.05.


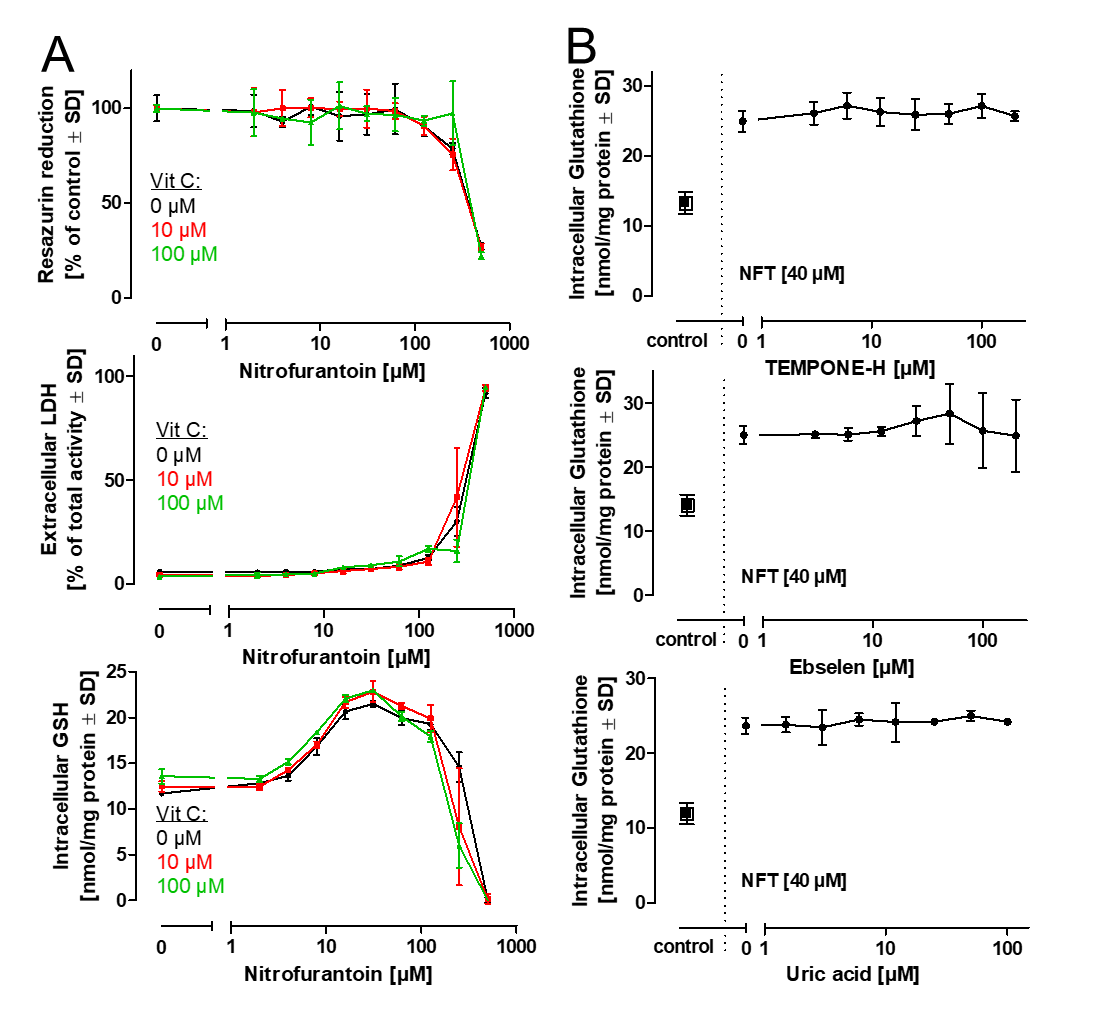


**Suppl. Fig. 10. Influence of free radical scavengers on NFT-dependent stimulation of glutathione synthesis.** ***A)*** HepG2 were treated with varying concentrations of NFT in the presence of different concentrations of ascorbic acid /vitamin C. ***B)*** HepG2 were treated with NFT (40 µM) in the presence of the spin-trap TEMPONE-H, the glutathione peroxidase mimic ebselen, or the peroxynitrite-scavenger uric acid. “control” cells received neither antioxidants nor NFT. In all experiments, the antioxidants were added 1 h before NFT. Viability (***A***) and glutathione (***B***) were measured after an incubation period of 48 h.


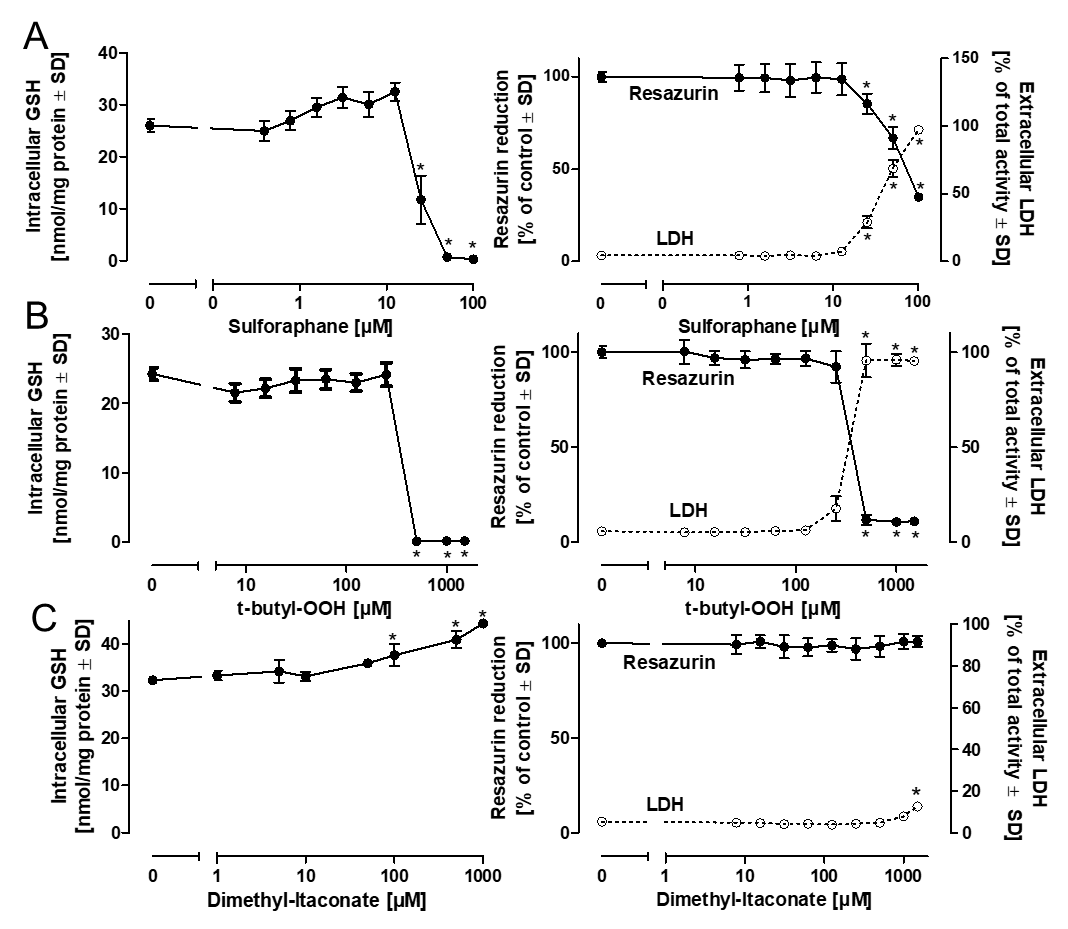


**Suppl. Fig. 11. Influence of Nrf2 activators on glutathione synthesis.** HepG2 were incubated with sulforaphane, t-butyl-OOH, or dimethyl-itaconate for 48 h and analyzed for the total content of glutathione. In parallel, viability was assessed by the resazurin reduction and by the lactate dehydrogenase release assays.
